# PIGO‐CDG: A case study with a new genotype, expansion of the phenotype, literature review, and nosological considerations

**DOI:** 10.1002/jmd2.12396

**Published:** 2023-09-20

**Authors:** Rodrigo Tzovenos Starosta, Nino Kerashvili, Cassandra Pruitt, Matthew J. Schultz, Suzanne W. Boyer, Eva Morava, Maria Laura Duque Lasio, Dorothy K. Grange

**Affiliations:** ^1^ Division of Genetics and Genomic Medicine, Department of Pediatrics Washington University in St. Louis Clayton Missouri USA; ^2^ Division of Pediatric Neurology, Department of Neurology Washington University in St. Louis Clayton Missouri USA; ^3^ Division of Academic Pediatrics, Department of Pediatrics Washington University in St. Louis Clayton Missouri USA; ^4^ Department of Laboratory Medicine and Pathology Mayo Clinic Rochester Minnesota USA; ^5^ Department of Clinical Genomics Mayo Clinic Rochester Minnesota USA; ^6^ Division of Laboratory and Genomic Medicine, Department of Pathology and Immunology Washington University in St. Louis Clayton Missouri USA

**Keywords:** congenital disorder of glycosylation, diarrhea, glycophosphatidylinositol, hyperphosphatasia, hypoglycemia, Mabry syndrome

## Abstract

The phosphatidylinositol glycan anchor biosynthesis class O protein (PIGO) enzyme is an important step in the biosynthesis of glycosylphosphatidylinositol (GPI), which is essential for the membrane anchoring of several proteins. Bi‐allelic pathogenic variants in *PIGO* lead to a congenital disorder of glycosylation (CDG) characterized by global developmental delay, an increase in serum alkaline phosphatase levels, congenital anomalies including anorectal, genitourinary, and limb malformations in most patients; this phenotype has been alternately called “Mabry syndrome” or “hyperphosphatasia with impaired intellectual development syndrome 2.” We report a 22‐month‐old female with PIGO deficiency caused by novel *PIGO* variants. In addition to the Mabry syndrome phenotype, our patient's clinical picture was complicated by intermittent hypoglycemia with signs of functional hyperinsulinism, severe secretory diarrhea, and osteopenia with a pathological fracture, thus, potentially expanding the known phenotype of this disorder, although more studies are necessary to confirm these associations. We also provide an updated review of the literature, and propose unifying the nomenclature of PIGO deficiency as “PIGO‐CDG,” which reflects its pathophysiology and position in the broad scope of metabolic disorders and congenital disorders of glycosylation.

**Synopsis**

We expand the phenotype of a patient with phosphatidylinositol glycan anchor biosynthesis class O protein (PIGO) deficiency to include right choanal stenosis, hypoglycemia, secretory diarrhea, and a pathological fracture, and propose the common nomenclature of “PIGO‐congenital disorder of glycosylation (CDG)” instead of “Mabry syndrome” or “hyperphosphatasia with impaired intellectual development syndrome 2.”

## 1 INTRODUCTION

The glycosylphosphatidylinositol (GPI)‐anchor biosynthesis defects are rare inborn disorders of the metabolism of complex molecules caused by deficiencies in different enzymes of this assembly pathway. The pathway starts with the insertion of a phosphatidylinositol group into the endoplasmic reticulum (ER) membrane, with subsequent synthesis of a glycan core composed of glucosamine and mannose and decorated with phosphoethanolamine moieties. Proteins can then be anchored to the terminal phosphoethanolamine moiety, and the GPI‐anchored protein complex is transferred from the ER to the Golgi apparatus, where the membrane insertion is remodeled. The mature GPI‐anchored proteins are then transferred to the outer leaflet of the plasma membrane, where they become part of lipid raft domains. To date, more than 150 proteins are known to be associated to lipid rafts through a GPI anchor, including enzymes, extracellular adhesion proteins, cell membrane receptors, immune proteins, and others.^1^, ^2^

The phosphatidylinositol glycan anchor biosynthesis class O protein (PIGO) is the enzyme responsible for attaching the terminal phosphoethanolamine residue to the third mannose residue of the GPI glycan core.^2^, ^3^ Loss of PIGO function through bi‐allelic pathogenic variants in the *PIGO* gene leads to a severe inborn error of metabolism first reported in 2012^4^ and characterized in most patients by severe global developmental delay, focal neurological disease (such as seizures or movement disorders), and congenital malformations of which the most common is Hirschsprung disease. To date, 18 patients with confirmed PIGO deficiency have been published in the scientific literature, and the genotypic and phenotypic spectrum of this disorder is still subject to expansion. We present a new case of PIGO deficiency caused by previously unreported compound heterozygous *PIGO* variants and displaying a severe phenotype.

### 1.1 Case report

**FIGURE 1:**
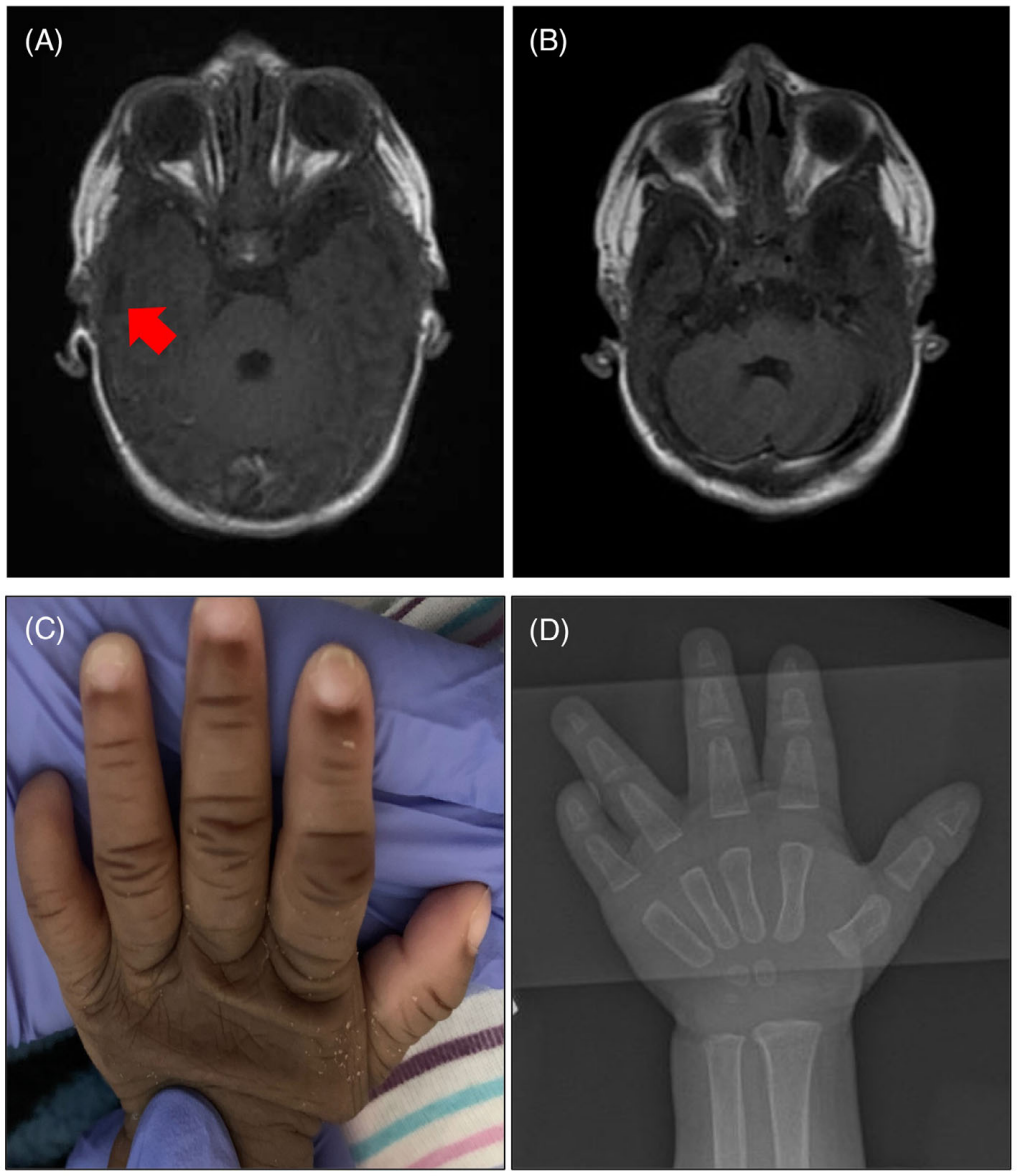
Brain MRI T1 sequences (obtained at 1 month of age). (A) Left temporal cyst. (B) Enlargement of the fourth ventricle. (C) Upper view of left hand showing wrinkled skin, nail dystrophy of digits 1–4, and nail aplasia of digit 5. This feature was present bilaterally. (D) Radiograph of left hand showing an absent distal phalanx of the 5th finger. This feature was present bilaterally.

The patient is a 22‐month‐old female who was born at term from nonconsanguineous parents of African American ancestry after a pregnancy complicated by maternal Crohn disease and by an abnormal prenatal ultrasound showing a small cerebellum. Birth parameters were within normal limits, including weight of 3290 g (54th percentile, WHO chart), length of 49 cm (43th percentile, WHO chart), and head circumference of 33 cm (22nd percentile, WHO chart). Her neonatal course was remarkable for failure to pass meconium in the first 3 days of life. Hirschsprung disease was diagnosed by contrast enema and suction rectal biopsy, and she underwent a pull‐through surgery on the day of life 4. After surgery, the patient was unable to tolerate reintroduction of breast or bottle feeds. A swallow study was notable for the aspiration of thin liquids. Head CT scan showed a right choanal stenosis without complete atresia. A gastrostomy tube (G‐tube) was placed in the first month of life. Brain MRI performed at 3 weeks of age showed a left temporal lobe cyst (Figure 1A) and a prominent fourth ventricle (Figure 1B). A neonatal hearing screen by auditory brainstem response showed bilateral sensorineural hearing loss.

At 2 months of age, the patient presented to the emergency room with discomfort with feeds leading to 12 h of decreased G‐tube intake. Physical exam showed microcephaly (head circumference 36 cm (second percentile, WHO chart), marked appendicular hypertonia with constant choreiform movements, and disconjugate eye movements. A routine electroencephalogram (EEG) was concerning for mild encephalopathy with nonspecific background changes. Blood glucose was 29 mg/dL (normal 70–199 mg/dL), prompting administration of dextrose‐containing fluids and admission to the hospital. Other laboratory tests obtained were remarkable for an elevated alkaline phosphatase level of 929 U/L (reference range 110–320 U/L). Upon reintroduction of G‐tube feeds, large‐volume emesis and abdominal distension were noted; rectal irrigations and dilations were attempted but were unsuccessful. A colonoscopy and an exploratory laparoscopy showed torsion of the pull‐through colonic segment requiring colonic resection and placement of an ileostomy. Physical exam was notable for large eyes, sparse scalp hair, bilateral preauricular ear pits, a long philtrum, bilateral clinodactyly of the 5th fingers with absence of the respective fingernails, as well as absence of the bilateral 4th and fifth toenails (Figure 1C,D). Chromosomal microarray (CMA, performed by the Genomic and Pathology Services at Washington University in Saint Louis, St. Louis, MO) and trio (patient and both parents) exome sequencing (ES) with mitochondrial sequencing and deletion analysis (performed by GeneDx LLC, Gaithersburg, MD) were obtained. CMA was negative. ES was significant for two variants of uncertain significance (VUSs) in *PIGO* (NM_032634.3):c.410C>T p.(Ala137Val), maternally inherited, and c.839T>C p.(Met280Thr), paternally inherited. No mitochondrial DNA sequencing or deletion variants were detected. The patient was discharged at 4 months of age on a stable G‐tube feeding regimen.

Subsequent clinical course was complicated by multiple admissions for feeding intolerance with abdominal distension and weight loss, as well as progressive worsening of choreiform movements and hypertonia. She was treated with gabapentin, clobazam, and clonidine with partial improvement in muscle tone and abnormal movements. EEGs showed nonspecific background abnormalities; multiple abnormal movements were captured without evidence of seizure activity electrographically. She was tried on different enteral formula regimens with a perceived better tolerance to a soy‐based regimen.

**FIGURE 2:**
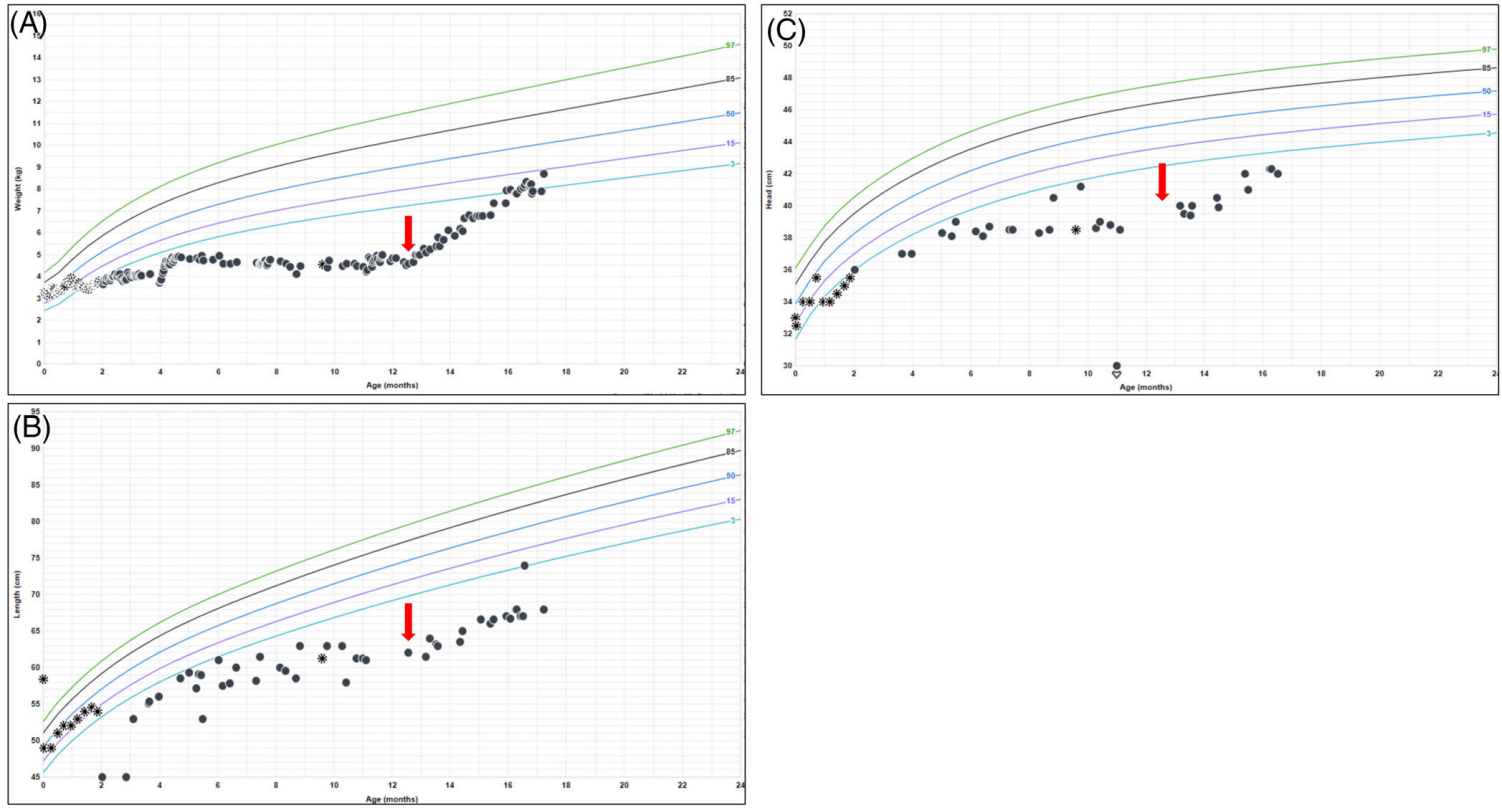
Growth curves. (A) Weight‐per‐age chart (World Health Organization). The red arrow represents initiation of chronic TPN. (B) Length‐per‐age chart (World Health Organization). The red arrow represents initiation of chronic TPN. (C) Head circumference‐per‐age chart (World Health Organization). The red arrow represents initiation of chronic TPN.

At 11 months of age, the patient started on outpatient total parental nutrition due to a lack of weight gain. This led to an admission due to an infection of her peripherally inserted central catheter (PICC) through which the TPN was administered, requiring treatment with multiple antibiotics. During this critical illness, intermittent hypoglycemia was noted (lowest blood glucose: 34 mg/dL, reference range: 70–199 mg/dL) although the patient was receiving IV fluids at a glucose infusion rate (GIR) of 12 mg/kg/min. Critical laboratory tests obtained during a hypoglycemic episode (blood glucose: 57 mg/dL) were remarkable for an inappropriately detectable insulin level of 1.8 μU/mL (reference range: 2.6–25.0 μU/mL), mildly low blood CO_2_ level of 18 mM (reference range: 20–30 mM), and inappropriately normal blood cortisol level of 6.0 μg/dL (reference range: 4.8–19.5 μg/dL). Blood beta‐hydroxybutyrate levels were undetectable, and blood lactate levels were normal at 0.7 mM (reference range: 0.7–2.0 mM). Given concern for functional hyperinsulinism, TPN was increased to a GIR of 19 mg/kg/min until resolution of hypoglycemia. The patient was discharged at 12 months of age on a stable GIR through TPN of 16 mg/kg/min, with a total caloric intake of 140 kcal/kg/day. She had marked improvement in growth parameters after the initiation of TPN (Figure 2).

At 14 months of age, the patient was admitted for a hemoglobin of 6.8 g/dL (reference range: 10.5–13.5 g/dL) and dyspnea. She required multiple blood transfusions for the resolution of her anemia. During that admission, she was noticed to have a prolapse of her ileostomy with a concern for incarceration, requiring a resection of approximately 15 cm of terminal ileum. During this admission, she had worsening of her choreiform movements, with an increase in her blood creatine kinase (CK) levels to 1139 U/L (reference range: ≤300 U/L). CK normalized after fluid resuscitation. At 18 months of age, she was again admitted for hypoxemia and dehydration secondary to an increase in ostomy output (approximately 70 mL/kg/day) despite not receiving enteral feeds. She was given daily IV pyridoxine at 20 mg/kg due to concern for spells of repetitive movements, which were not confirmed as seizures on EEG. She received a course of octreotide 2.5 μg/kg every 8 h for 10 days with no significant change in ostomy output during administration, and it was discontinued; however, there was an eventual gradual decrease in ostomy output over the subsequent 2 weeks to approximately 5 mL/kg/day at the time of discharge. She was discharged on nasal oxygen due to continuing sporadic desaturations.

The patient was most recently admitted at 19 months of age due to respiratory distress with an increase in her home oxygen requirements. She was found on a chest CT to have bilateral lower lobe bronchiectasis and bronchial wall thickening, with scattered atelectasis. She was started on a high‐flow nasal cannula. Due to a concern for pain with mobilization a radiographic skeletal survey was obtained, showing diffuse osteopenia and a healing Salter‐Harris type II fracture of the left distal tibia and fibula. Vitamin D, calcium, and phosphorus levels were normal. The fracture was casted, and the patient was started on IV bisphosphonate therapy with zoledronic acid. She is currently 23 months old.

### 1.2 Mass cytometry

Whole blood from the patient and from two concurrently collected adult donors was washed, barcoded, and stained with the Fluidigm MaxPar Direct immunoprofiling kit. Multiplexed samples were acquired on a Helios CyTOF mass cytometry instrument (Standard BioTools, South San Francisco, CA) from the same analysis tube. Granulocytes were identified using canonical markers and CD16 expression determined by the amount of heavy metal tag bound to cells Data analysis and plotting were performed in R version 4.2.1.

**FIGURE 3:**
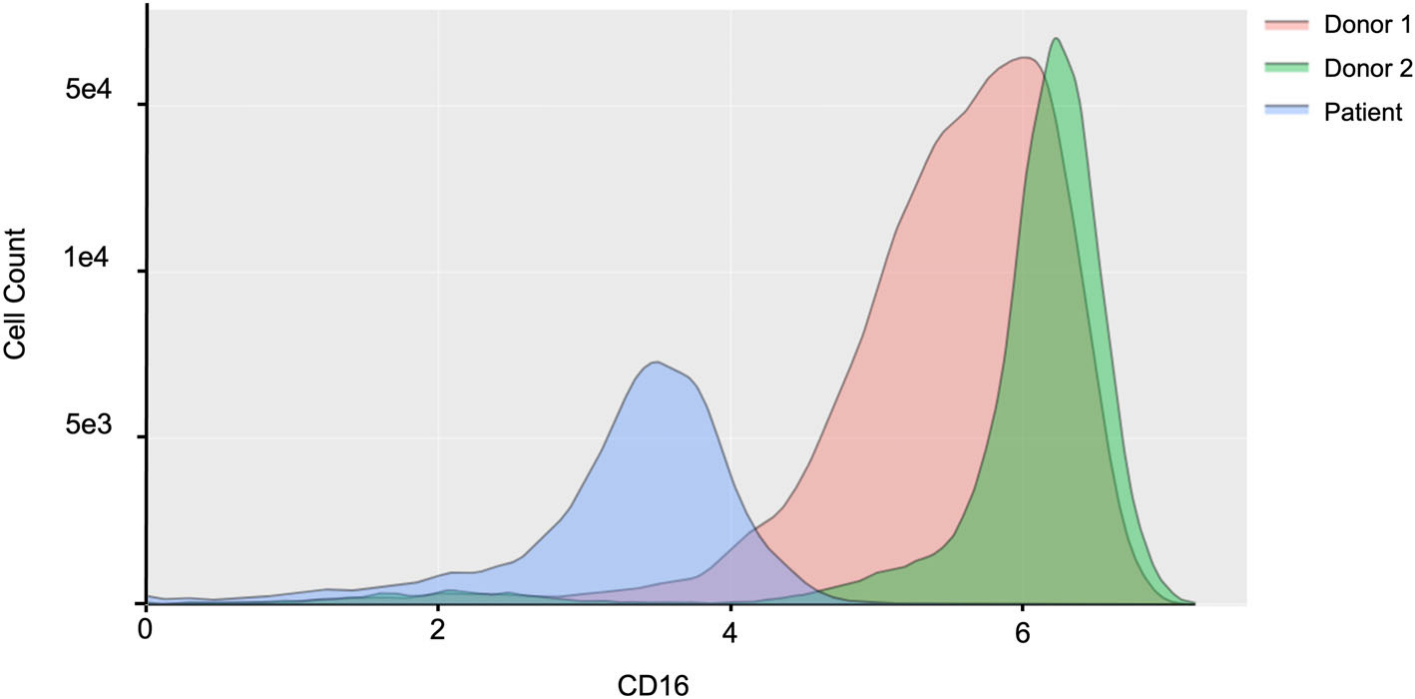
Mass cytometry results for CD16 expression in circulating granulocytes. The expression of CD16 in the patient's granulocytes, as well as granulocyte abundance, is markedly decreased when compared to two healthy controls.

Analysis of patient cells by mass cytometry assay demonstrated abnormalities consistent with decreased GPI‐AP expression on the cell surface. CD16 expression on granulocytes was reduced compared to two intratube donor samples run as controls (Figure 3).

### 1.3 Variant analysis

**FIGURE 4:**
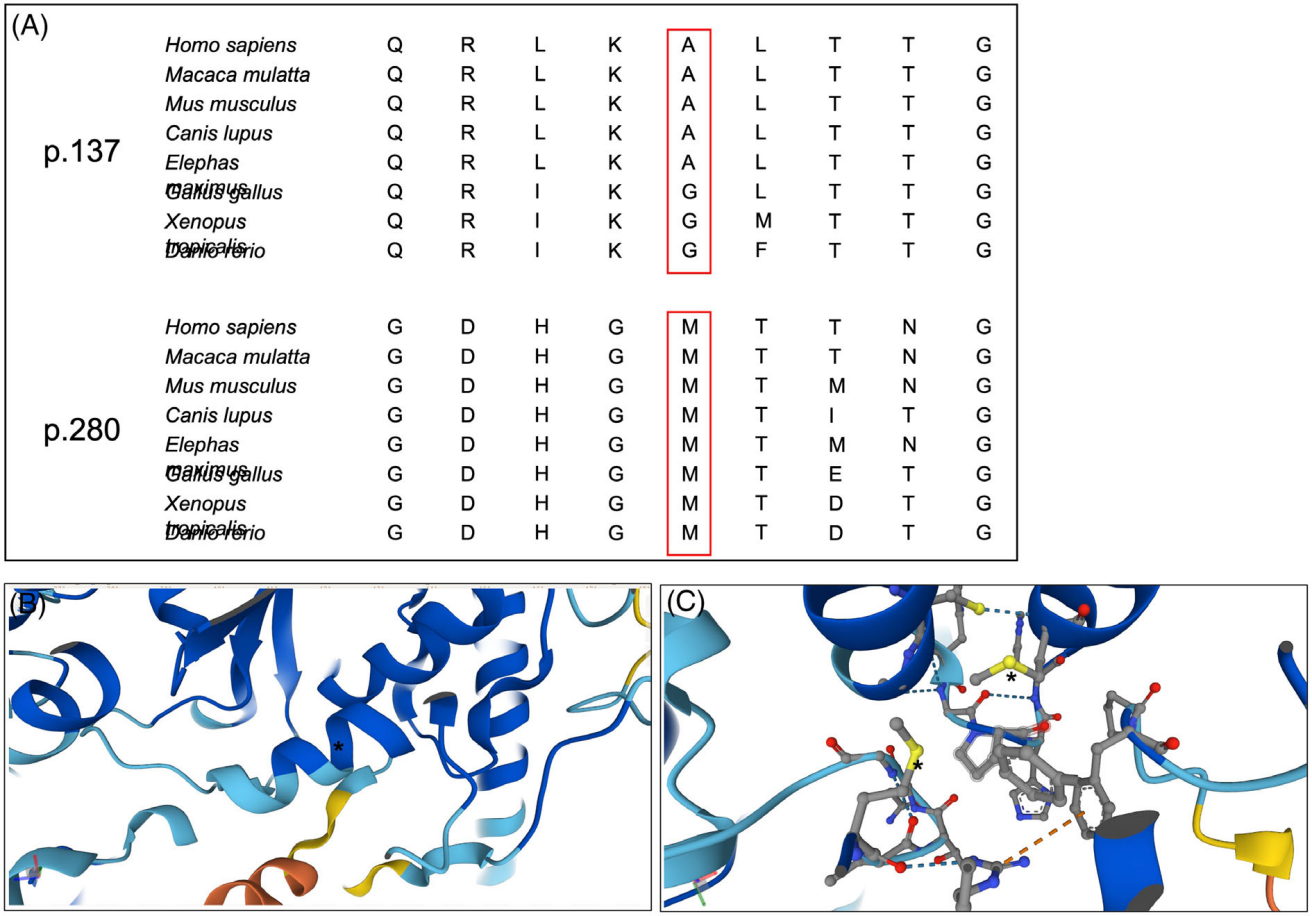
Variant analysis. (A) Evolutionary conservation comparison for the residues that are variant in our patient. The red box highlights the variant residues. (B) Structural protein view of PIGO (created by AlphaFold^32^, ^33^). The asterisk (*) highlights Ala137. (C) Structural protein view of PIGO (created by AlphaFold^32^, ^33^). The asterisks (*) highlight the sulfur atoms of Met280 (left) and Met248 (right).

The c.410C>T p.(Ala137Val) variant, which was inherited from the patient's mother, is located at an α‐helix in the transferase domain of PIGO. This variant substitutes a valine, which is a weak α‐helix destabilizing amino acid, for an alanine, which is a standard helix stabilizing amino acid^5^ evolutionarily conserved in this position in mammals (Figure 4A,B). There is in silico consensus for deleteriousness (Table 1). This variant meets the American College of Medical Genetics/Association for Molecular Pathology (ACMG/AMP) variant classification criteria PM2_m, PP3_m, PP4_m, and can thus be classified as likely pathogenic.^6^

The c.839T>C p.(Met280Thr) variant, which was inherited from the patient's father, is located in a hinge region between a beta‐sheet and an alpha‐helix. This variant changes a nonpolar residue to a polar one, and likely disrupts a methionine‐methionine interaction between Met280 and Met248 (Figure 4C). This residue is conserved from humans to zebrafish, as are all other residues N‐proximal to it in this hinge, potentially reflecting an important and delicate function in maintaining the spatial structure of this protein. This variant meets the ACMG/AMP variant classification criteria PM2_m, PP3_m, PP4_m, and can thus be classified as likely pathogenic.^6^

## 2 DISCUSSION

An autosomal recessive disorder associated with pathogenic variants in *PIGO* was first described by Krawitz et al. in 2012^4^ as “hyperphosphatasia with mental retardation syndrome (HPMRS),” currently renamed as “hyperphosphatasia with impaired intellectual development syndrome 2 (HIIDS2)” (MIM #614749). In 2010, Thompson et al. named a similar condition of GPI biosynthesis defect “Mabry syndrome” based on the clinical description of five patients with similar dysmorphic features, persistent hyperphosphatasia, digital abnormalities, and neurodevelopmental abnormalities,^7^ matching a phenotype first described by Mabry et al. in 1970.^8^ Currently, 18 patients have been reported in the medical literature as having Mabry syndrome/HIIDS2 with confirmed bi‐allelic pathogenic variants in *PIGO*.^4^, ^9^, ^10^, ^11^, ^12^, ^13^, ^14^, ^15^ While all reported patients have global developmental delay with intellectual disability (which is often in the severe range), other clinical features are variable and include (summarized in Table 2): seizures (12/18), intestinal malformations such as Hirschsprung disease and anal atresia (11/18), digital abnormalities including brachydactyly and nail hypoplasia (9/18), sensorineural hearing loss (5/18), chorea (3/18), congenital genitourinary malformations (3/18), congenital heart disease (3/18), and esophageal atresia (2/18). Craniosynostosis, cleft palate, and vertebral abnormalities were observed in one patient each. Dysmorphic facial features were reported in 10/18 and most often include wide palpebral fissures, a prominent nasal bridge, and a long and smooth philtrum. Hyperphosphatasia (i.e., an elevated serum alkaline phosphatase level) was present in 13/18 individuals. Neuroimaging findings often include white matter lesions (5/18), cerebellar atrophy (4/18), cerebral atrophy with ventriculomegaly (4/18), a thin corpus callosum (3/18), diffusion restriction in the basal ganglia or brainstem (3/18), and hypoplastic optic nerves and chiasm (1/18). Decreased expression of GPI‐anchored proteins in circulating granulocytes has been reported in several patients and is considered one of the biochemical hallmarks for this disorder, although there is variability in the reduction of different markers.^4^, ^10^, ^12^, ^14^, ^16^

We present a female patient with facial dysmorphic features, right choanal stenosis, digital abnormalities with nail hypoplasia, chronic hyperphosphatasia, severe global developmental delay, hypertonia, chorea, congenital bilateral sensorineural hearing loss, Hirschsprung disease, and a clinical course complicated by dehydration from a high ostomy output, hypoglycemia with functional hyperinsulinism and hypocortisolism, and rhabdomyolysis; mass cytometry analysis of peripheral granulocytes showed a significant reduction of CD16. While the core features of this case fit the classic phenotypic description by Mabry et al.,^8^ some manifestations expand the known phenotype of this disorder and merit further consideration. One of the major clinical challenges in this patient has been the difficulty with the management of her fluid status due to increased ostomy output in the setting of illnesses despite the lack of enteral nutrition. Although individuals that have undergone colectomies are expected to have some loss of free water and electrolytes through their ostomies due to the lack of free water resorption by the colon, the ostomy output seen in this patient has been significantly higher than the 10–15 mL/kg/day described in this population.^17^ The severe feeding intolerance with lack of weight gain despite numerous enteral regimens is also striking and, in conjunction with a possible decreased intestinal water reabsorption, may point to a generalized small bowel mucosal dysfunction caused by the deficiency of GPI‐anchored proteins in this patient. We hypothesize that a possible mechanism for this intestinal failure may include loss of glycocalyx‐associated proteins at the enteral mucosa, such as loss of glypicans and glypican‐bound heparan sulfate at the mucosal capillaries leading to increased extravasation of fluid from the intravascular to the intestinal luminal compartment.^18^, ^19^ Glycocalyceal dysfunction has been previously suggested as a pathogenic mechanism in a cohort of patients with congenital disorders of glycosylation, including a patient with PIGN‐CDG reported by Brucker et al. for whom fresh frozen plasma and protein C concentrate infusions were given, with the rationale of supporting the endothelial glycocalyx during acute illnesses, with excellent results.^20^ It is possible that further studies, especially using the recently validated murine model for PIGO deficiency,^21^ will better delineate the role of the glycocalyx and the etiology of intestinal dysfunction in these patients and provide therapeutic insights for addressing this issue.

To the best of our knowledge, this is the first time that hypoglycemia has been reported as a feature of GPI‐anchor biosynthesis disorders. The presence of hypoglycemia while the patient was receiving IV dextrose at a high GIR as well as an inappropriately incomplete suppression of insulin point to a functional hyperinsulinemic state, likely exacerbated by overall illness and malnutrition. The fact that hypoglycemic episodes were only detected after prolonged fasting or during periods of physiological stress, and that it has not been previously reported as a feature of these disorders, may be an indication that GPI‐anchor deficiency does not directly lead to functional hyperinsulinism. It is possible that this disorder decreases instead the ability of the pancreas to dynamically regulate insulin secretion in response to noxious stimuli and thus only predisposes patients to hypoglycemia during illness or fasting. Regardless of the specific mechanism for hypoglycemia, our case highlights the importance of proactively monitoring blood glucose levels in these patients, especially during periods of intercurrent illness.

The cause of neurological dysfunction in PIGO deficiency is still subject to investigation. One of the main theories focuses on the role of decreased activity of GPI‐anchored alkaline phosphatases, especially tissue nonspecific alkaline phosphatase (TNAP). TNAP is a multi‐function, multi‐site enzyme with broad dephosphorylating activity, including the conversion of the hydrophilic form of vitamin B6 pyridoxal‐5′‐phosphate into pyridoxal, which can cross the blood–brain barrier into the central nervous system (CNS).^22^, ^23^, ^24^ Among its functions in the CNS, pyridoxal acts as a co‐factor for glutamate decarboxylase, which synthesizes γ‐aminobutyric acid (GABA), one of the main inhibitory neurotransmitters in the CNS, from glutamate; decreased GPI‐anchored TNAP, therefore, has been suggested to lead to GABA deficiency, contributing to the pathogenesis of epilepsy in patients with GPI‐anchor deficiencies.^12^, ^23^ In the clinical setting, this hypothesis was tested by pyridoxine supplementation for seizure control in patients with different GPI‐anchor biosynthesis disorders, with clinical and electrographic response achieved in some individuals.^16^, ^25^ The lack of complete response and the fact that seizure burden was unchanged in some individuals, however, likely points to a multifactorial etiology of epilepsy in this population. We observed no significant improvement of our patient's choreiform movements while on pyridoxine. We hypothesize that GABA dysfunction may also contribute to the pathogenesis of chorea in our patient, and hope that experimental studies will be able to further elucidate this aspect in the future.

The nomenclature of PIGO deficiency and the other GPI biosynthesis disorders is currently not organized systematically, which can cause confusion for caregivers. The “Mabry syndrome” designation, while a good description of the common phenotypical characteristics in these patients, does not allow for a precise etiological differentiation between patients, since it is applied to conditions caused by pathogenic variants in several different genes; this may lead to problems when genotype–phenotype correlations are discovered.^4^, ^11^ The “HIIDS2” designation, on the other hand, while connecting the disease with the underlying genetic etiology (as each number in the “HIIDS” phenotypic series is associated with a different enzyme in the GPI biosynthesis pathway), focuses the description on hyperphosphatasia, which is not present in all patients with the disorder^12^, ^14^ and, as discussed previously, does not seem to be the only pathogenic factor for its core features.^14^ In the past decade, Jaak Jaeken and colleagues have proposed a new classification of the congenital disorders of glycosylation (CDG), expanding the definition of a CDG from defects only in N‐glycosylation enzymes to defects that affect the building and modulation of different types of glycans (including specialized glycolipids such as GPI).^26^, ^27^ This was reflected in the most recent consensus classification of inborn errors of metabolism to include the GPI biosynthesis defects and other lipid glycosylation disorders as a subgroup of the CDGs, which are included in the category of defects of complex molecule metabolism.^28^ We propose unifying the nomenclature of PIGO deficiency as “PIGO‐CDG.” This emphasizes the underlying biochemical defect and its status as a metabolic disorder affecting the processing of complex molecules. This also allows for uniformity with other GPI‐anchor defects named according to Jaeken et al.,^26^ such as PIGA‐CDG,^29^ PIGN‐CDG,^30^ or PIGT‐CDG.^31^ Given the large phenotypic overlap with N‐linked glycosylation disorders, this facilitates the inclusion of caregivers in parent‐organized associations for support and sharing of experiences, as we have seen with the parents of our patient.

**TABLE 1:** Interpretation of the *PIGO* variants.

	c.410C>T p.(Ala137Val)	c.839 T>C p.(Met280Thr)
Parent of origin	Mother	Father
CADD score^34^	25.7	26.2
PolyPhen‐2 prediction^35^	Probably damaging (0.981)	Probably damaging (0.999)
SIFT prediction^36^	Damaging	Damaging
GnomAD frequency^37^	3.18 × 10^−5^	3.98 × 10^−6^
GnomAD heterozygotes	0	0
ACMG/AMP classification	PM2_m, PP3_m, PP4_m	PM2_m, PP3_m, PP4_m
Interpretation	Likely pathogenic variant	Likely pathogenic variant

Abbreviations: ACMG/AMP, American College of Medical Genetics/Association for Molecular Pathology; CADD, combined annotation dependent depletion; PIGO, phosphatidylinositol glycan anchor biosynthesis class O protein; SIFT, sorting intolerant from tolerant.

**TABLE 2:** Comparison of phenotypical features reported in the literature and in the present case.

Phenotypic feature	Literature (*n* = 18)	Present case
Global developmental delay	++++	Present
Hyperphosphatasia	+++	Present
Seizures	+++	Absent
Hirschsprung disease/anal atresia	+++	Present
Digital abnormalities	+++	Present
Dysmorphic features	+++	Present
Neuroimaging findings	+++	Present
Sensorineural hearing loss	++	Present
Movement disorders	++	Present
Genitourinary malformations	++	Absent
Congenital heart disease	++	Absent
Esophageal atresia	++	Absent
Craniosynostosis	+	Absent
Cleft palate	+	Absent
Vertebral malformations	+	Absent
Choanal stenosis	−	Present
Hypoglycemia	−	Present
Secretory diarrhea	−	Present
Pathological fracture	−	Present

*Note*: ++++ = present in all; +++ = present in 50% or more; ++ = present in less than 50% but in more than one; + = present only in one; and − = absent;

## 3 CONCLUSION

We present a complex patient with a diagnosis of PIGO‐CDG complicated by severe failure to gain weight and global developmental delay, Hirschsprung disease requiring total colonic resection leading to multiple admissions for dehydration secondary to an increased ileostomy output, choreiform movements, and hypoglycemia during illnesses with signs of functional hyperinsulinism. Our patient has required multiple admissions and complex care coordination. We have discussed the expansion of the phenotype associated with this disorder to include hypoglycemia and chronic diarrhea. We reviewed the current gaps in knowledge of the physiopathology leading to the core associated features. Finally, we propose the nomenclature of “PIGO‐CDG” instead of “Mabry syndrome” or “HIIDS2,” which may allow for better characterization of this disorder and a better understanding of the diagnosis by caregivers and healthcare professionals.

## AUTHOR CONTRIBUTIONS

Rodrigo Tzovenos Starosta followed the patient clinically, classified the variants, reviewed the literature, and wrote the first draft of the manuscript. Nino Kerashvili, Cassandra Pruitt, Suzanne W. Boyer, Eva Morava, Maria Laura Duque Lasio followed the patient clinically and provided essential input into the manuscript. Matthew J. Schultz performed the mass cytometry analysis and provided essential input into the manuscript. Dorothy K. Grange diagnosed and followed the patient clinically and provided essential input into the manuscript. All authors reviewed and approved the submitted version of this manuscript.

## FUNDING INFORMATION

National Institute of Neurological Disease and Stroke, Grant/Award Number: 1U54NS115198‐01; National Center for Advancing Translational Sciences; Rare Disorders Consortium Disease Network.

## CONFLICT OF INTEREST STATEMENT

The authors declare no conflict of interest.

## ETHICS STATEMENT

All procedures followed were in accordance with the ethical standards of the responsible committee on human experimentation and with the Helsinki Declaration of 1975, as revised in 2000.

## INFORMED CONSENT

The patient's parents gave their informed consent for the publication of this case report.

## Data Availability

Data and material were accessible through institutional medical records at the respective institutions.
